# Seed biology and regeneration niche of the threatened cold desert perennial *Ivesia webberi* A. Gray

**DOI:** 10.3389/fpls.2025.1568951

**Published:** 2025-05-05

**Authors:** Israel T. Borokini, Michael D. France, Daniel Harmon, Kevin T. Shoemaker, Peter J. Weisberg, Mary M. Peacock

**Affiliations:** ^1^ Program in Ecology, Evolution and Conservation Biology, Department of Biology, University of Nevada, Reno, Reno, NV, United States; ^2^ Department of Ecology, Montana State University Bozeman, Bozeman, MT, United States; ^3^ Great Basin Rangeland Research Laboratory, United States Department of Agriculture, Reno, NV, United States; ^4^ Department of Natural Resources and Environmental Science, University of Nevada, Reno, Reno, NV, United States; ^5^ Department of Biology, University of Nevada, Reno, Reno, NV, United States

**Keywords:** *Ivesia webberi*, seed viability, germination rate, multispectral imaging, cold stratification, physiological dormancy

## Abstract

Understanding the regeneration niche is of critical importance for the conservation of rare plants, yet species-specific information is often lacking for key components of the plant life cycle such as seed dormancy and germination. We conducted a detailed study of the regeneration niche for *Ivesia webberi*, a U.S. federally threatened forb that is endemic to the Great Basin Desert. Using seeds collected from 11 populations across a span of years, we investigated seed storage behavior, embryo morphology, and interannual and interpopulation seed viability, while testing the efficacy of alternative nondestructive methods to assess seed viability. We also studied the effects of various pre-incubation and incubation treatments on germination rates, speed, and synchrony. An examination of x-ray images showed that *I. webberi* have non-endospermic seeds with spatulate embryos. We observed a significant reduction in seed viability over three years, suggesting a recalcitrant storage behavior. Seed viability exhibited significant interannual, but not interpopulation, variation across 11 *I. webberi* populations. Both the x-ray and multispectral imaging are promising nondestructive methods that can replace the widely used, but destructive, tetrazolium test. Across all 68 germination treatments, seed germination was higher, faster, and more synchronized under warmer cold-stratified incubation temperatures. Seed germination was significantly increased by pre-incubation chilling and reduced by pre-incubation heat treatments, while pre-incubation and incubation light exposures had no effect. Both the seed embryo morphology and germination experiments suggest physiological dormancy in *I. webberi*. Results suggest that warmer and shorter winters, such as are consistent with predicted climate change, could increase germination of *I. webberi* seeds.

## 1 Introduction

The seed is an important stage in the plant life cycle. It determines regeneration, recruitment of new individuals into a population, dispersal and new colonization events and gene flow for many plants (Li et al., 2018; Infante-Izquierdo et al., 2020; Chen K. et al., 2022); therefore, rates of seed mass evolution are strongly associated with speciation rates in angiosperms (Igea et al., 2017). Thus, understanding the regeneration niche, that is, various biotic, genetic, climatic factors that drive flowering, pollination, seed production, dormancy, dispersal, germination, and seedling establishment (Grubb, 1977; Rosbakh et al., 2018), is important for predicting plant population demography under global changes and post-disturbance recovery (Rosbakh et al., 2018; Glison et al., 2023). Regeneration niche studies can also be used to predict phenology shifts under changing climate (Footitt et al., 2018; Vázquez et al., 2024), and to identify factors driving high mortality rates during the transition from seed to seedling and across seedling life stages, as well as their impacts on recruitment (Young et al., 2005; Jiménez-Alfaro et al., 2016; Valdez et al., 2019).

Seed dormancy is an adaptation strategy to ensure optimal germination in favorable conditions (Baskin and Baskin, 2014). Conditions that favor seed germination vary widely among plants, depending on the type of dormancy, storage time, distribution ecology, embryo morphology, and mating system, among others (Kildisheva et al., 2020; Chen J.-Z. et al., 2022). Germination requirements are highly species-specific (James et al., 2020; Verhoeven et al., 2024). For example, over 70% of alpine plants require cold stratification and light for seed germination (Schwienbacher et al., 2011; Fernández-Pascual et al., 2021), whereas, desert plants need water and temperature increases for seed dormancy release (Baskin and Baskin, 2014). Some desert plants germinate under broad dormancy-releasing treatments, while spring germinators need cold stratification for optimal germination (Forbis, 2010). Some plant species require fire or chemical treatment in the gut of herbivores to break dormancy (Cosyns et al., 2005; Milotić and Hoffman, 2016; Lamont et al., 2019). Understanding the conditions associated with dormancy release can optimize successful translocation for threatened species and can be used to reliably predict how plant regeneration and seedling recruitment would be impacted by global changes (Copete et al., 2005; Herranz et al., 2010).

Conservation scientists and managers have leveraged seed dormancy for seed banking purposes. With over 1700 seed banks in the world, seed banking is the oldest and most common *ex situ* conservation strategy for species management and global food security (Food and Agriculture Organization, 2010; Hay and Probert, 2013; Potter et al., 2017; Díez et al., 2018; Liu et al., 2018). Archived and conserved germplasms can then be used for post-disturbance vegetative community regeneration, translocation of threatened species to suitable habitats, as well as *de novo* crop propagation (Engels and Ebert, 2021). Investigating the potential of seed banks to manage wild populations of threatened species is particularly warranted as such banks historically have focused on plants of agricultural significance (Merritt and Dixon, 2011; Meyer et al., 2014; Abeli et al., 2019). A further conservation challenge exists for species that produce recalcitrant seeds and hence may not be suitable for seed banking (Berjak and Pammenter, 2008; Wyse et al., 2018; Wyse and Dickie, 2018), comprising up to 10% of all angiosperms and about 40% of species on the IUCN Red List of Threatened Species.

Maintaining the viability of stored seeds is pivotal to successful *ex situ* conservation; for example, studies showed that 38% of plant re-introductions from seed banks were partially successful, while 31% failed completely (Abeli et al., 2019). Thus, monitoring seed viability is essential in managing conservation seed banks. One major limitation is that seed stocks of rare plants may be too low for the periodic application of destructive methods such as tetrazolium or seedling emergence tests (Abeli et al., 2019). Therefore, there is a strong need for seed viability testing methods that are both reliable and nondestructive (Baek et al., 2019). Non-destructive seed testing methods, such as seed x-ray and multispectral imaging, reveal seed properties that are indirectly used to infer seed viability. Seed x-rays can also be used to visualize seed development, embryo morphology, and potential pest and pathogenic damage from which inferences are drawn about seed health, viability, and storage behavior (Gagliardi and Marcos-Filho, 2011; Costa et al., 2014). Likewise, multispectral imaging can be used to assess seed health, moisture level, purity, fruit maturity, and detect pest damage (Vrešak et al., 2016; Boelt et al., 2018; Baek et al., 2019).

**Figure 1:**
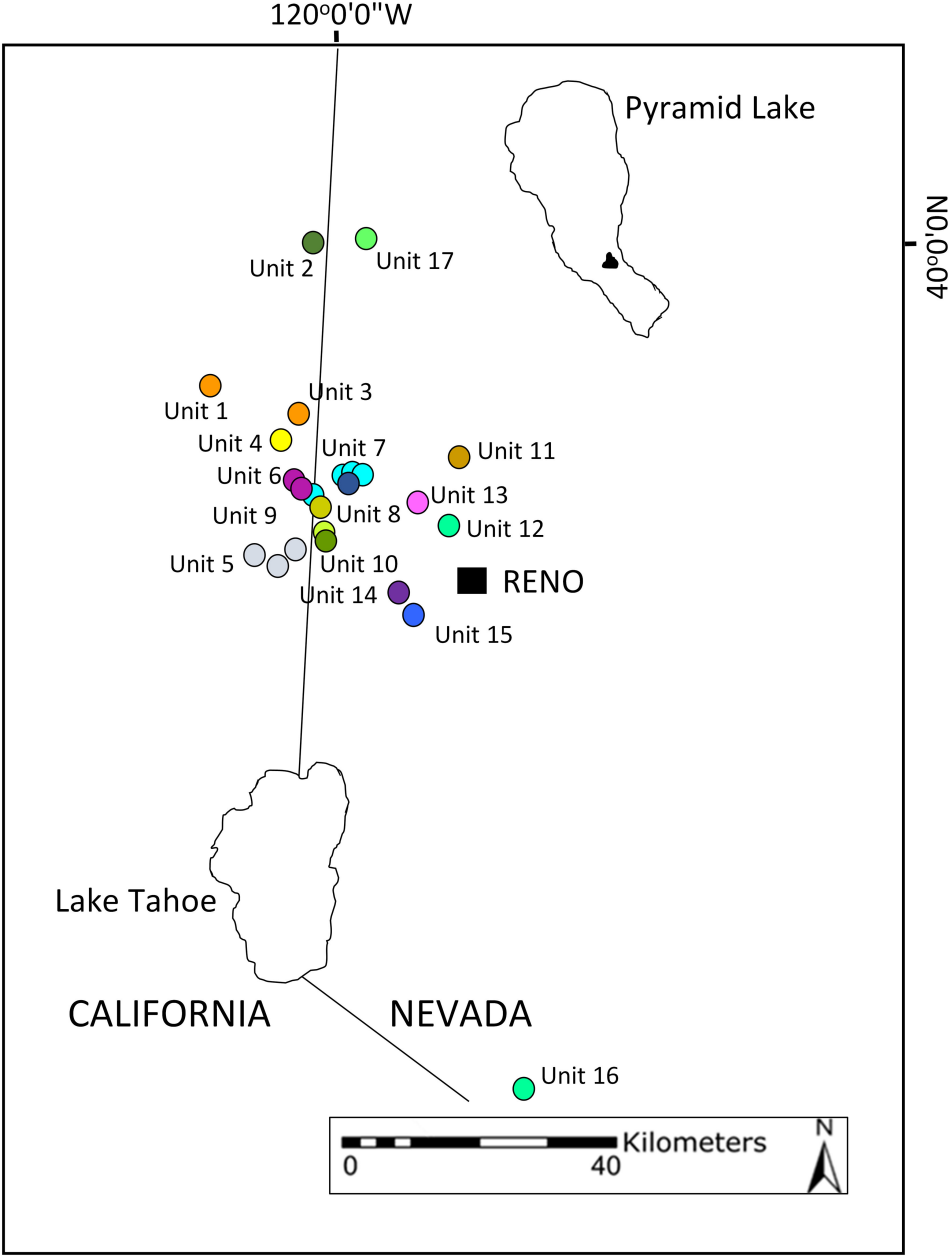
Global distribution of *Ivesia webberi* populations. Unit numbers follow the USFWS designations, circles represent the geographic center of extant, mapped occurrences, and circles with same color indicate USFWS-designated subpopulations of the same population.

In this study, we described seed embryo morphology and investigated seed viability and germination of *Ivesia webberi* A. Gray (Webber’s Ivesia, or wire mousetail), a U.S. federally threatened perennial herb belonging to the Rosaceae family. This species has a narrow distribution in the *Artemisia arbuscula* steppe in the western Great Basin Desert and northeastern foothills of the Sierra Nevada Range and is currently found in 32 locations (**Figure 1**) (Witham, 2000; Borokini et al., 2023). We asked the following specific questions: (a) Do *I. webberi* seeds lose their viability over time under ambient storage conditions? (b) Is there a significant interannual and interpopulation variability in *I. webberi* seed viability? If so, what proportion of this variation is explained by climatic variables? (c) Can non-destructive methods accurately predict viability of *I. webberi* seeds? (d) What treatments enhance seed germination success and speed and improve synchrony of *I. webberi* seed germination? (e) How will the predicted mild winter and warmer spring seasons affect *I. webberi* seed germination? An understanding of seed germination processes in *I. webberi* will support management and conservation of this federally threatened species.

## 2 Materials and methods

### 2.1 Ivesia webberi

*Ivesia webberi* regenerates in late winter or early spring, both vegetatively from dormant root caudices and from seed recruitment, which are produced from a mixed mating system characterized by both selfing and outcrossing (USFWS, 2014; Borokini et al., 2021a). The species produces yellow capitate or sub-capitate cyme inflorescences containing between five and 15 flowers on each flowering stalk, which when fertilized, develop into light brown colored, dry indehiscent achenes (Witham, 2000). The seeds are small, between 1.9 and 2.5 mm, smooth and mottled, and between three and eight seeds are produced per flower (Witham, 2000). However, seed dispersal is localized within rock crevices that characterize the soil surface in all population sites (USFWS, 2014; Witham, 2000). From field observations, there is no evidence to suggest significant seed predation on *I. webberi*. Patch sizes vary widely among known locations (**Figure 1**; **Table 1**) and are impacted by invasive species and wildfires (USFWS, 2014; Borokini et al., 2021b). Seedling emergence and age-class structure were reported from field observations (Witham, 2000), but drought spells and invasion by non-native weeds may impact natural seedling recruitment (Borokini et al., 2021b). Moreover, local experts reported limited success in germinating *I. webberi* seeds, suggesting the likely importance of seed dormancy for this species.

**Table 1:** Location, site, and population characteristics, and mean viability of the seed collections from 11 *Ivesia webberi* population sites in the western Great Basin Desert, United States.

Unit^a^	Site location	County and State	Site area (m^2^)^b^	Abundance estimate^c^	Sample size 2017	Mean ± SE viability 2017	Sample size 2018	Mean ± SE viability 2018
2	Near Constantia	Lassen CA	7,700	100-999	31**	0.20 ± 0.07	40	0.00 ± 0.00
3	East of Hallelujah Junction	Lassen CA	1,400	115-130	31***	0.68 ± 0.09	39	0.62 ± 0.08
5	Dog Valley Meadows	Sierra CA	289,700	100,000	25**	0.64 ± 0.10	45	0.53 ± 0.08
6	White Lake Overlook	Sierra CA	54,900	10,000	30*	0.47 ± 0.09	45	0.64 ± 0.07
7	Mules Ear flat	Sierra CA	1,400	<100	27	0.33 ± 0.09	35	0.83 ± 0.06
8	Ivesia flat	Washoe NV	3,000	100,000	27	0.44 ± 0.10	26	0.46 ± 0.10
11	Hungry Valley	Washoe NV	600	2,120	33	0.15 ± 0.06	38	0.63 ± 0.08
12	Black Springs	Washoe NV	25,500	>500-1000	31*	0.52 ± 0.09	45	0.69 ± 0.07
13	Raleigh Heights	Washoe NV	38,600	<100,000-4,000,000	30	0.23 ± 0.08	44	0.66 ± 0.07
14	Dutch Louie flat	Washoe NV	5,500	600,000-693,795	30	0.07 ± 0.05	41	0.68 ± 0.07
16	Dante Mine Road	Douglas NV	2,300	3,179-36,500	30	0.23 ± 0.08	43	0.70 ± 0.07

^a^USFWS unit designation for the *I. webberi* populations (see USFWS, 2014); ^b^Site area was calculated from USFWS (2014); ^v^Abundance estimate for each population was sourced from USFWS (2014). ***p < 0.001, **p < 0.01, *p < 0.05 following results from the logistic regression to investigate statistical difference in seed viability across sampled populations in 2017 and 2018. The viability of seeds collected in 2018 was not significantly different across sampled populations.

### 2.2 Seed viability analyses

#### 2.2.1 Seed viability tests

Three seed viability tests were used in this study: (1) the standard 2,3,5 triphenyl tetrazolium chloride test (hereafter referred to as tetrazolium or TZ test); (2) X-ray imaging; and (3) multispectral imaging. The TZ test is recognized by the Association of Official Seed Analysts and the International Seed Testing Association as a highly precise and accurate test of seed vigor (Nurse and DiTommaso, 2005; Gosling et al., 2009; de Barros França-Neto and Krzyzanowski, 2019). Seeds were imbibed in water, cut, and soaked in tetrazolium solution. Healthy and live seeds produce hydrogen ions, from the activity of dehydrogenase enzymes, which reduces colorless tetrazolium to red triphenyl formazan; the resulting red color indicates seed viability (de Barros França-Neto and Krzyzanowski, 2022). All TZ tests were carried out at the Idaho State Seed Laboratory, Boise, Idaho, United States.

**Figure 2:**
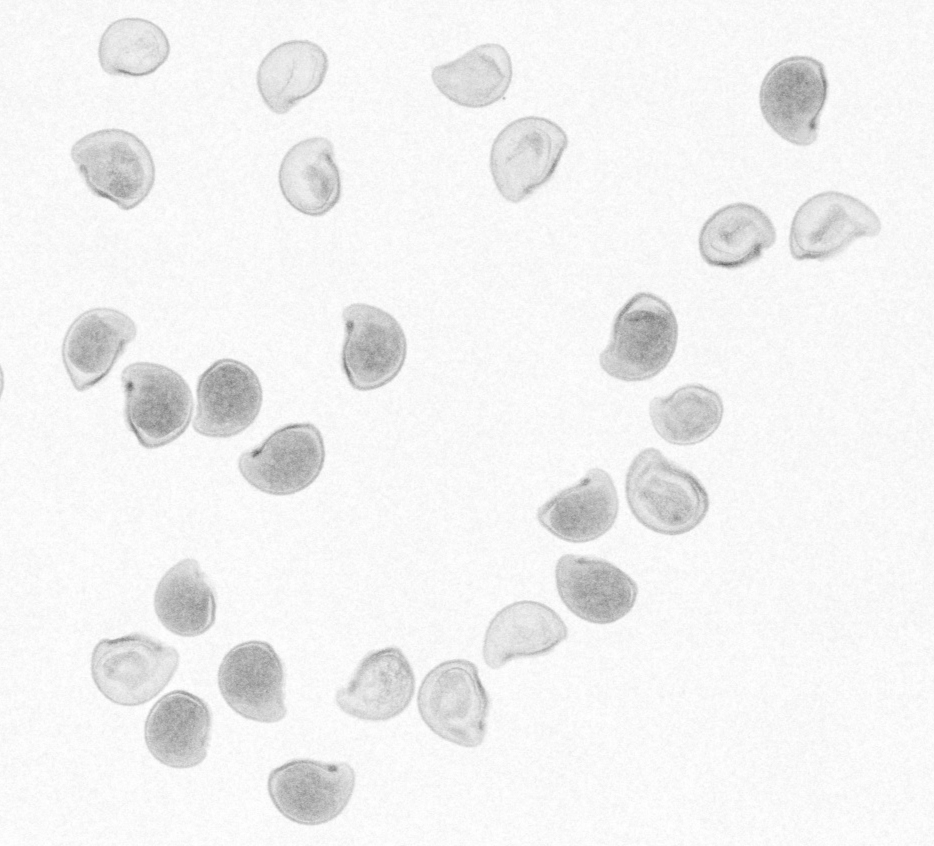
Plate of x-ray imagery of *Ivesia webberi* seeds showing filled and unfilled embryos. Shaded seeds represent filled seeds indicating matured embryo, while unfilled seeds are considered empty with immature or no embryo.

X-ray imaging was conducted at the United States Forestry Service (USFS) Bend Seed Extractory, Bend, Oregon, following methods described in Gomes et al. (2016). X-ray images for each seed were captured at a radiation intensity of 26 kV for 1.2 seconds, using a digital Kubtec medical imaging Xpert 40 specimen radiography system. A visual inspection of the seed x-ray images was used to discriminate between viable and nonviable seeds. Seeds with dark shadows in the x-ray images are indicative of filled and matured embryos and were scored as viable (**Figure 2**). Conversely, seeds with light or no shading in the x-ray images were considered nonviable (**Figure 2**). Additionally, the seed x-ray imagery allowed us to examine the internal seed tissues and describe the seed embryo morphology, following published seed classification standards (Martin, 1946; Atwater, 1980; Ellis et al., 1985).

Multispectral imaging was conducted at Skyway Analytics LLC, Longmont, Colorado (https://getskywayanalytics.com/). Each seed was placed in a 90 mm petri dish without cover, and digital images were captured with a VideometerLab 3 instrument (Halkjaer Olesen et al., 2011; Su and Sun, 2018). The multispectral images of 1280 × 960 pixels were captured at 26 different spectral bands, covering the visible (380–780 nm) and near-infrared (780–2500 nm) regions (Huang et al., 2015; Boelt et al., 2018), to describe seed testa chemical and spectral properties. Additionally, seed size, width, length, shape, orientation, and color were also measured for each seed.

#### 2.2.2 Effect of storage time on the viability of *Ivesia webberi* seeds

*I. webberi* seeds were collected from the Unit 5 population in August 2017, 2018, and 2019, when matured seeds were ready for abscission (**Figure 1**). We used this population because it is the largest (**Table 1**), thus minimizing the potential effect of seed collection pressure on *I. webberi* populations. Empty seeds were identified and removed using the finger pressure test. Remaining healthy seeds (n = 50, 45, 50 for 2017, 2018, and 2019, respectively) were stored in coin envelopes, under ambient conditions of 21°C and 15% humidity. The healthy seeds collected in 2017, 2018, and 2019 were stored for two, one year, and three months, respectively, following which TZ test was performed on seeds from each storage time category. The viability (0 = non-viable, and 1 = viable) of individual seeds collected between 2017 and 2019 was modeled as a function of storage time, treated as a categorical variable with three levels: 0, 1 and 2 years in storage, using logistic regression. A Tukey’s HSD test was used to perform *post-hoc* pairwise comparisons (Abdi and Williams, 2010).

#### 2.2.3 Interannual and population-level differences in the viability of *I. webberi* seeds

Between 50 and 100 seeds were collected from 11 *I. webberi* populations of varying patch sizes (**Table 1**), in August of 2017 and 2018. Healthy seeds from these collections were stored under ambient conditions of 21°C and 15% humidity, for eight months in coin envelopes. Storing the seeds for several months before viability testing was done to allow the seeds an after-ripening period for full embryo development if necessary (Baskin and Baskin, 2014). A post-abscission ripening period is common for winter and spring annual and perennial plants (Chantre et al., 2009; Forbis, 2010). Due to limitations in seed collection from threatened species and many empty seeds, sample sizes varied across sampled populations for the 2017 and 2018 collections (**Table 1**). We conducted the TZ test on the seeds collected for this experiment. We conducted logistic regression models and Tukey’s HSD *post-hoc* multiple comparisons to investigate the effect of patch sizes on *I. webberi* seed viability. We also conducted student’s t-test to investigate variation in the viability of seeds collected in 2017 and 2018.

To investigate the effect of climatic conditions on *I. webberi* seed viability across the two years of collection (2017 and 2018), we calculated seasonal actual and potential evapotranspiration (AET and PET, respectively), climatic water deficit (CWD), and annual water content (AWC), heatload and topographic variables (elevation, slope, and cosine aspect) for 2017 and 2018. AET, PET, CWD, AWC and heatload were calculated using the Thornthwaite water balance model (Lutz et al., 2010; Dilts, 2015; Dilts et al., 2015) based on monthly PRISM climatic averages (1971–2019 at 800 m resolution; PRISM Group, 2007), USDA SSURGO soil data (USDA, 2013), and a digital elevation model. Correlated variables were removed using Pearson correlation coefficient (-0.6 < *r* < 0.6) and the remaining predictor variables (summer AET 2017 and 2018, cosine aspect, slope, and heat load) were used to fit a multivariate multiple linear regression on the mean seed viability for 2017 and 2018 seed samples, following which Type II MANOVA Pillai *post-hoc* test was conducted.

#### 2.2.4 Estimating the reliability of non-destructive x-ray and multispectral imaging to discriminate between viable and non-viable *I. webberi* seeds

The total number of seeds (n = 441) collected in 2018 (described in 2.2.3 above) were used to investigate the potential of non-destructive seed testing methods (**Table 1**). X-ray images of the 441 seeds were taken first, followed by multispectral imaging and the TZ test. The 42 continuous variables derived from the multispectral imaging and binary scoring of the x-ray imageries were considered the predictor variables, while the binary scoring of the TZ test was used as the response variable. However, as large portions of the electromagnetic spectrum were likely to be redundant with respect to seed viability indicators, this resulted in unnecessary data multidimensionality (Chen Z. et al., 2014; Baek et al., 2019). Therefore, we used the variable reduction feature implemented in the Boruta R package (Kursa and Rudnicki, 2010) and the backward stepwise recursive feature elimination algorithm in the caret R package (Kuhn, 2008) to reduce the predictor variables to three predictor variables. These three uncorrelated variables – seed x-ray imagery, seed width, and seed spectral reflectance at 690 nm – were used to build the final model for seed viability.

We fitted a random forest classification model (ntree = 500, mtry = 2) to the three selected variables using the party R package (Hothorn et al., 2006) with supporting utility functions written by KTS. Variable importance was assessed as the loss of predictive accuracy (Gini statistic) when random permutations of each predictor variable were performed for randomly drawn samples (Cutler et al., 2007). Partial dependence plots were used to illustrate the relationship between each of the three predictors and seed viability (Friedman, 2001). We used a 10-fold cross validation to assess overall predictive performance (Cutler et al., 2007), using the area under the receiver operating characteristic curve (AUC; ROCR package in R; Sing et al., 2005) as the primary performance metric (Fielding and Bell, 1997).

### 2.3 Seed germination analyses

#### 2.3.1 Seed imbibition test

Seeds previously harvested in 2016 in the USFWS designated unit 7b (**Table 1**) and stored by the Nevada Department of Forestry were used for the seed germination experiments. First, we conducted a seed imbibition test to determine if the seed testa is permeable to water. Six replications of 50 healthy seeds were dried, weighed, and placed on moistened filter paper in petri dishes, while being kept at room temperature (Kildisheva et al., 2018). Seed weight was measured at time 0, representing initial seed mass (W_d_), and at 1, 2, 4, 8, 24, 48, 72 and 96-hour intervals. Measurement was stopped at 96 hours when seed germination was observed. Seeds were weighed to the nearest 0.001 g using a Sartorius CPA225D semi-micro digital analytical laboratory balance. Percentage mass increase (%W_s_), indicating seed weight increase, was calculated as:


$$\%{\text{W}}_{\text{s}}=[({\text{W}}_{\text{i}}–{\text{W}}_{\text{d}})/{\text{W}}_{\text{d}}]\times 100$$

where W_s_ = increase in seed mass, W_i_ = mass of seeds after a given interval of imbibition, and W_d_ = initial mass of seeds (Hidayati et al., 2000). The result of the imbibition test was shown as a plot of percentage seed mass increase over time.

#### 2.3.2 Seed germination experimental designs

Using a three-factorial experiment, we conducted seed germination trials testing the effect of pre-incubation and incubation temperature and light exposure on germination success, speed, and synchrony of *I. webberi* seeds. These pre-incubation and incubation treatments mimic natural conditions that *I. webberi* seeds are subjected to post-abscission from parent plant in summer and winter months, prior to germination in early spring (Forbis, 2010). A power analysis (df = 3 at *p* < 0.05 and model explanatory power of at least 50% of the variance in the data) indicated that the use of 100 seeds for each treatment is sufficient for the seed germination experiments. For each treatment, we had four replicates (petri dishes) of 25 seeds each.

In the first phase, *I. webberi* seeds were subjected to factorial treatment combinations of pre-incubation temperature [i.e., cold moist (1°C), warm dry, and warm moist exposure (30°C for 14 hours, and 15°C for 10 hours)] and light exposure (either 12-hour light exposure or complete darkness) for four weeks, following which the seeds were incubated under cold stratification (5°C for 12 hours and 1°C for 12 hours in a 24-hour day cycle) in either 12-hour light or 24-hour darkness (**Supplementary Table 1**, treatments 3-18). We included two controls, none of which underwent pre-incubation treatments, but were incubated under 12-hour light or 24-hour darkness (**Supplementary Table 1**, treatments 1-2). Additional treatments included factorial combinations of seeds soaked in different concentrations of gibberellic acids, potassium nitrate solutions, and a mixture of both growth hormones (**Supplementary Table 1**, treatments 19-34). Incubation by cold stratification is widely reported for germinating alpine and subalpine plants (Porceddu et al., 2013; Baskin and Baskin, 2014; Mondoni et al., 2015). We confirmed the importance of cold stratification for *I. webberi* in two trial germination investigations prior to this study. Light exposures were done with fluorescent lamps and a photosynthetic photon flux density of 19 to 22 mmol/m^2^/s, while seeds subjected to total darkness were covered with double layers of aluminum foil. All 34 treatment combinations were incubated for 12 weeks, while the petri dishes were kept continuously moist, and germination recorded weekly. A seed was considered to have germinated when radicle emergence, of at least 2 mm in length, was observed.

The second phase of seed germination experiments (**Supplementary Table 1**, treatments 35-68) was similar to the first phase, except that pre-incubation cold moist exposure was maintained at 2°C, while the 12-week incubation temperature was maintained at 15°C for 12 hours, and 2°C the remaining 12 hours, representing predicted climatic conditions of mild winter and warmer spring seasons. Moreover, 50 seeds were selected and subjected to TZ test before the first and second germination experiment phases to check for possible differences in seed viability, given that the second experiment phase started three months after the first phase ended. Seed germination experiments were conducted at United States Department of Agriculture (USDA) Agricultural Research Service (ARS) Seed Laboratory, Reno, Nevada.

#### 2.3.3 Effect of light vs darkness on *Ivesia webberi* seed germination

Two statistical analyses were conducted to test the effect of 12-hour incubation light exposure vs total darkness on seed germination. The bivariate data, containing germination of seeds exposed to 12-hour incubation light and those in total darkness, was subjected to relative light germination percentage (RLGP) test to evaluate light requirement for *I. webberi* seed germination (Milberg et al., 2000; Wang et al., 2009):


RLGP=Pl/(Pd+Pl),

where Pl is percentage germination in light, and Pd is percentage germination in darkness. RLGP ranges from 0 to 1 indicating preference for germination in darkness and light, respectively. Even though RLGP gives us a single value to compare germination success between light and dark treatments, it does not produce tests of significance. Therefore, we ran Fisher’s 2-proportion test of equality (Fisher’s Exact probability test) to test for significant difference in seed germination for 12-hour light and total darkness treatments. The Fisher Exact probability test is a non-parametric technique for comparing proportions, testing the null hypothesis that the probabilities of success in two groups are the same. Both the RLGP analysis and the Fisher’s Exact test were conducted separately for the first and second germination experiment phases and both phases combined.

#### 2.3.4 Effect of pre-incubation and incubation treatments on *Ivesia webberi* seed germination

Using the germination records from all pre-incubation and incubation treatments and controls (treatments 1-18, 35-52, **Supplementary Table 1**), we fitted separate generalized linear mixed models (GLMMs), holding incubation temperature and incubation light exposure, as random effects to investigate the effects of pre-incubation and incubation treatments on seed germination success. We also fitted baseline GLMMs including all 68 treatments to study the effects of the growth hormones used in the experiments, with incubation temperature and incubation light exposure as random effects. While our research questions focus on investigating the effects of treatments that mimic natural conditions (light and temperature), we used the baseline model as a reference and to test for the effects of growth hormones.

#### 2.3.5 Effects of pre-incubation and incubation treatments on *Ivesia webberi* seed germination time and synchrony

We investigated the effect of the 68 pre-incubation and incubation treatments on the timing of seed germination in *I. webberi*. Many species of perennial forbs growing in desert ecosystems experience shortened generation times and exhibit germination bet hedging strategies. Using functions implemented in GerminaR R package (Lozano-Isla et al., 2019), we calculated mean germination time (MGT) and synchronization index Z (**Supplementary Table 1**). The mean germination time is defined as the time required for the seeds to germinate during the experiments (Ranal et al., 2009; Lozano-Isla et al., 2019), and is calculated as:


MGT=∑(n×d)/N,

where n is the number of newly germinated seeds each day, d is the number of days from the beginning of the experiment, and N is the total number of germinated seeds at the end of the experiment (Ellis and Roberts, 1981). Germination synchronization index Z evaluates the degree of overlap in the germination of two seeds under the same treatment (Ranal et al., 2009; Lozano-Isla et al., 2019). Lower Z values indicate synchronized germination, while higher values indicate asynchronous germination, indicative of bet hedging strategy. We tested the effects of all pre-incubation and incubation treatments on mean germination time (MGT) and synchronization index Z (SYN), for the two germination experiment phases separately and collectively, using analysis of variance (ANOVA) tests. All data analyses were conducted in R statistical software and RStudio interface (R Core Team, 2024; RStudio Team, 2024).

## 3 Results

### 3.1 The effect of storage time on the viability of *I. webberi* seeds

**Figure 3:**
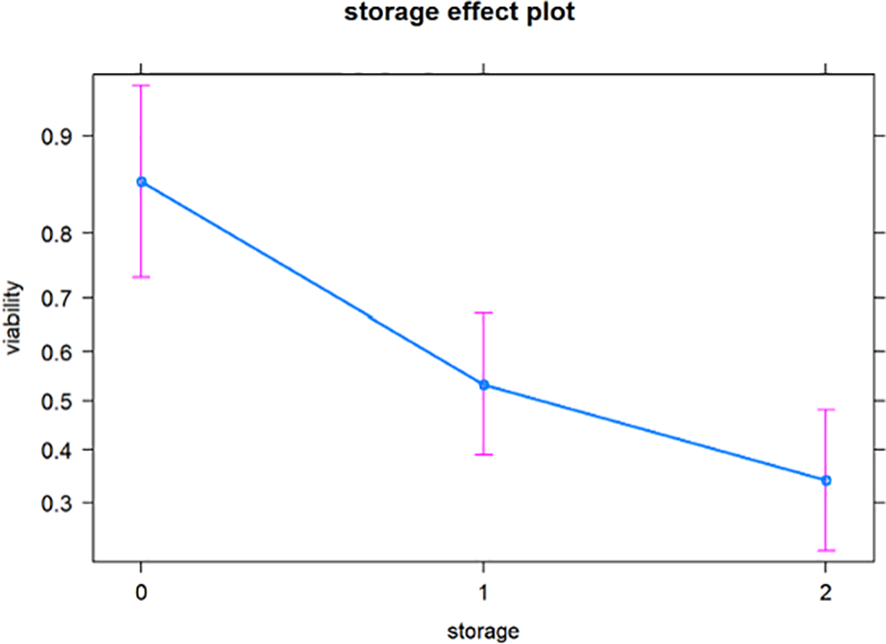
A plot of the predicted viability trends through time based on logistic regression for *Ivesia webberi* seeds stored between 2017 and 2019.

The viability of *I. webberi* seeds decreased with storage time (**Figure 3**); seeds stored for three months had 86% viability, while seeds stored for one and two years had 53% and 34% viability respectively. There were significant pairwise differences in seed viability between seeds stored for three months and those stored for one year (z = -3.33, *p* < 0.001) and two years (z = -4.91, *p* < 0.001) respectively.

### 3.2 Population-level and interannual difference in the viability of *I. webberi* seeds

The viability of seeds collected in 2017 showed variation among populations (χ2 = 45.0, df = 10, *p* < 0.001) with significant differences among sampled populations exhibiting the highest seed viability (units 3 and 5) and those with the lowest seed viability (units 2, 11, and 14; **Table 1**). However, the viability of *I. webberi* seeds collected in 2018 showed no significant differences among the 11 populations. This contrasting result for 2017 and 2018 may be attributed to interpopulation variability in seed viability, which was higher for the 2017 collections (mean = 0.36, SD = 0.48, CV = 135%) than for the 2018 collections (mean = 0.59, SD = 0.49, CV = 83.5%).

The viability of *I. webberi* seeds showed significant interannual variability (student’s t = -2.5, df = 19.9, *p* = 0.02) between 2017 and 2018. Broadly, seed viability was lower in 2017 than in 2018; for example, only three populations had ≥50% seed viability in 2017 collections, in contrast to nine populations in 2018 (**Table 1**). These significant differences could be attributed to an overall positive effect of summer 2017 AET (Pillai test statistic = 0.87, F = 13.83, *p* = 0.02) and negative effect of heatload (Pillai test statistic = 0.85, F = 10.99, *p* = 0.03; see **Supplementary Table 2**) on the two-year seed viability.

### 3.3 Reliability of seed testa spectral properties and x-ray imagery to predict *I. webberi* seed viability

**Figure 4:**
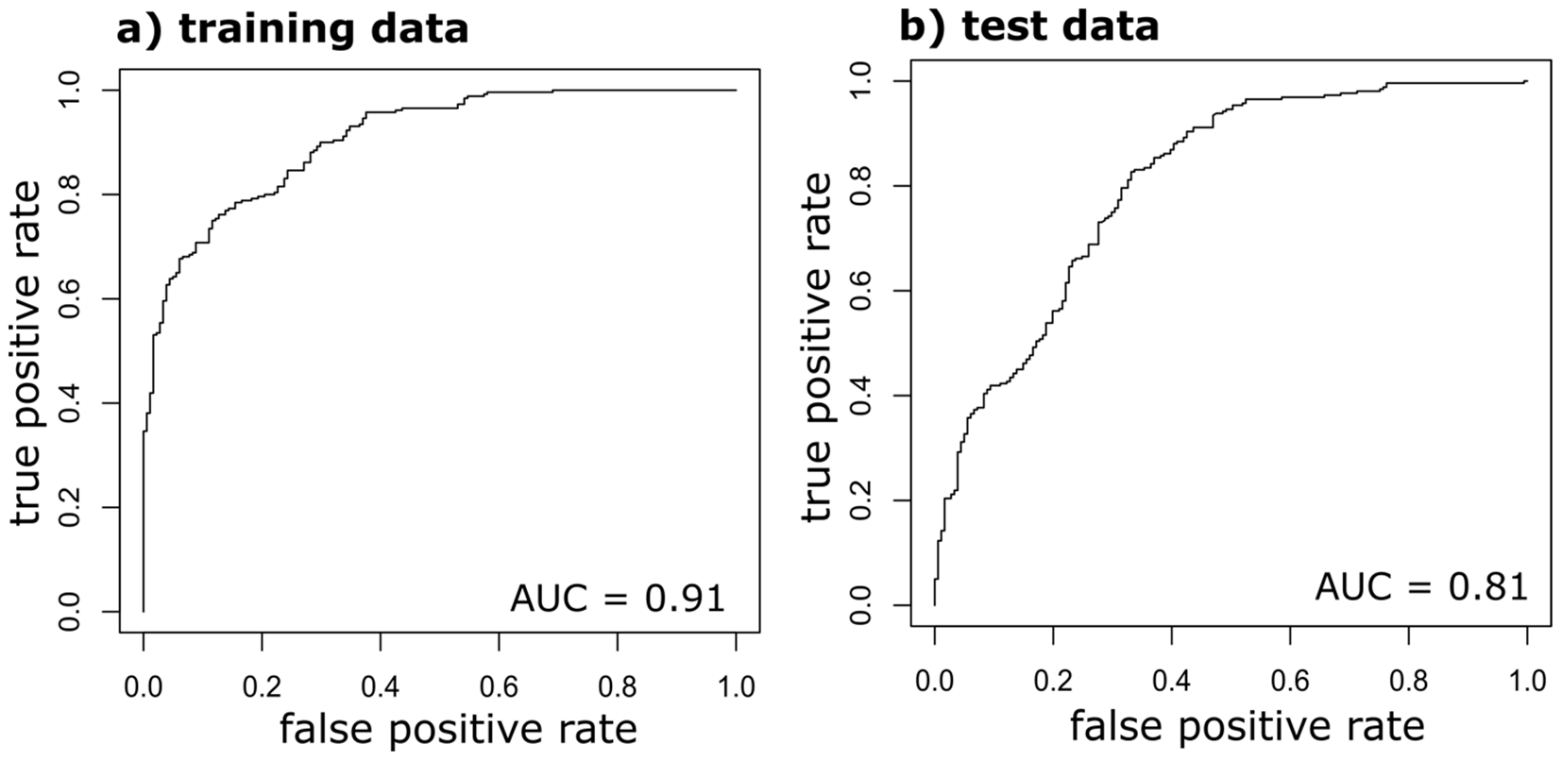
A receiver operating characteristic (ROC) plot showing the area under curve (AUC) of the random forest model training **(a)** and test **(b)** data for non-destructive *Ivesia webberi* seed viability classification.

**Figure 5:**
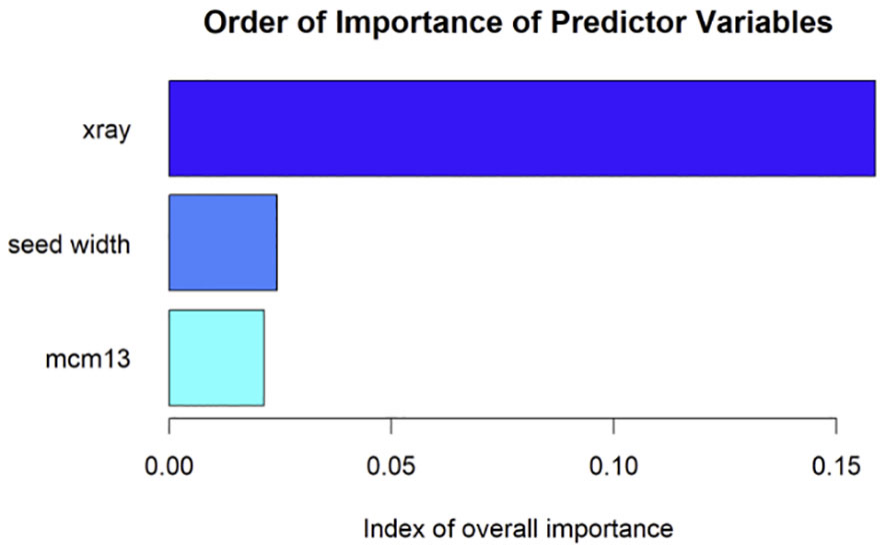
A plot of the relative contributions of the three predictor variables on the random forest model predicting *Ivesia webberi* seed viability. MCM13 represents seed testa spectral reflectance at 690 nm.

**Figure 6:**
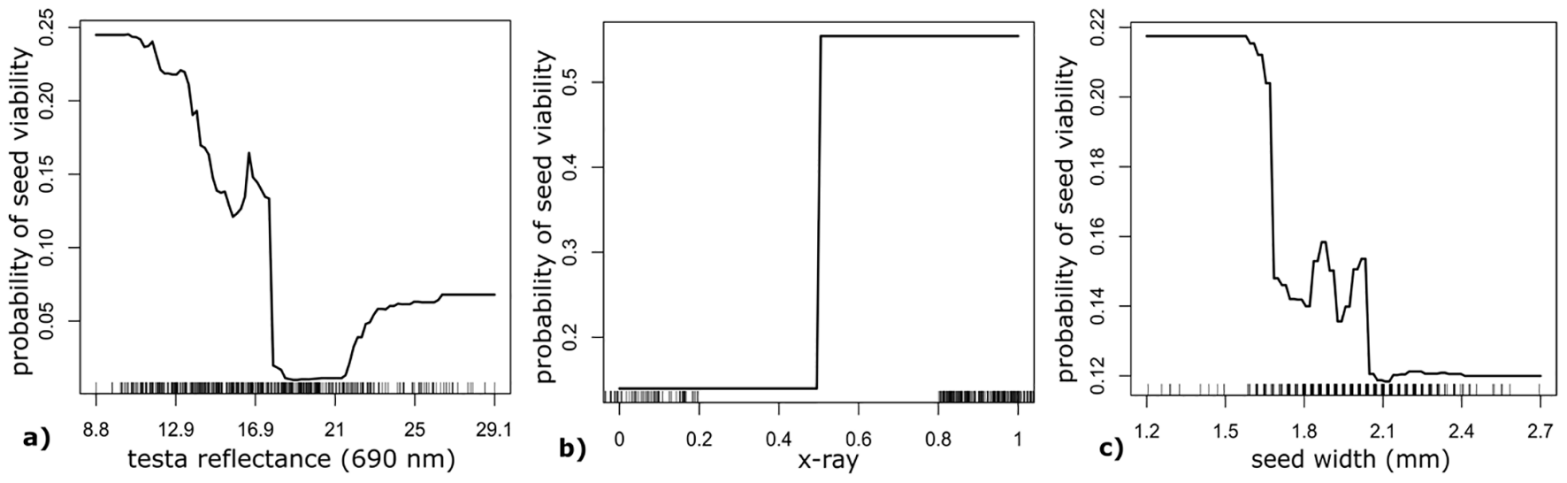
Univariate plots showing seed viability for **(a)** seed testa reflectance at 690 nm, **(b)** seed x-ray, and **(c)** seed width computed from a random forest model for non-destructive *Ivesia webberi* seed viability classification.

TZ test results showed 260 of the 441 individual seeds collected in 2018 as viable. Simple t-tests for viable and nonviable seeds conducted between mean values for seed x-ray, seed width, and spectral reflectance at 690 nm were significantly different at *p* < 0.01 (**Supplementary Table 3**). The Random Forest model had high model performance and prediction (accuracy = 0.82, specificity = 0.93, AUC_train_ = 0.91, AUC_test_ = 0.81; **Figures 4a, b**). Seed x-ray imagery contributed the most to the model, followed by seed width and 690 nm seed spectral reflectance (**Figure 5**). Univariate partial dependence plots showed that the probability of *I. webberi* seed viability increases with decreasing seed testa spectral reflectance at 690 nm (**Figure 6a**), filled seeds in the x-ray imagery (**Figure 6b**) and lower seed width values (**Figure 6c**). Moreover, a significant inverse relationship between seed area and viability for seeds collected in 2018 was observed, although this relationship was nonsignificant for the 2017 seed collections (see **Supplementary Result 1**; **Supplementary Figure 1**).

### 3.4 Seed imbibition test

**Figure 7:**
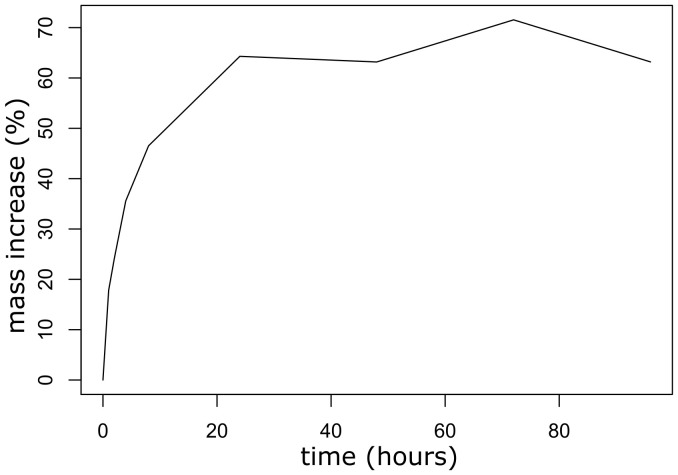
A plot of percentage seed weight increases during a 96-hour imbibition test of *Ivesia webberi* seeds.

Within a few hours of soaking seeds in water, the seed weight increased, indicating water penetration and absorption through the seed testa (**Figure 7**), suggesting that mechanical or chemical scarification is not required for seed dormancy release.

### 3.5 Assessment of light requirement for *I. webberi* seed germination

We recorded 419 and 372 seed germinations under light and dark treatments, respectively for the first experiment phase (5/1°C). The second phase (15/2°C) resulted in 498 and 522 germination counts for light and dark treatments, respectively. However, the Relative Light Germination Percentage (RLGP) analysis showed no distinct light requirement for seed germination in *I. webberi*. RLGP values were 0.52 and 0.49 for seed germination experiments under 5/1°C and 15/2°C incubation temperatures, respectively. Overall, RLGP was 0.51 for both phases of seed germination experiments combined. There was no significant difference (*p* > 0.05) between seed germination counts for experiments under light or darkness for both experimental phases, thus supporting the RLGP results.

### 3.6 Effects of pre-incubation and incubation treatments on *I. webberi* seed germination

In the first experimental phase, with an incubation temperature of 5/1°C, we recorded 791 germinations out of 3400 seeds while the second phase with 15/2°C incubation temperature resulted in 1020 seed germinations. The generalized linear mixed model (GLMM; incubation temperature as random effect) showed that all pre-incubation treatments (light exposure, chilling temperature, and heat treatment) except incubation light, had significant effects (*p* < 0.05) on seed germination (**Table 2**). Among all pre-incubation treatments, *I. webberi* seeds chilled for four weeks produced the highest germination, while seeds subjected to heat treatments performed poorly in both germination phases (**Supplementary Table 1**).

**Table 2:** Results of the generalized linear mixed model for *I. webberi* seed germination subjected to varying pre-incubation light (12-hour light vs 24-hour darkness), either cold moist (1 or 2°C), warm dry or warm moist (30/15°C), and either 12-hour incubation light exposure or 24-hour darkness, while accounting for incubation temperature difference (5/1°C or 15/2°C) between the two experiment phases, as a random effect.

Factor	Estimates	Standard error	z-value	*P*
Intercept	-0.76	0.15	-5.16	<0.01
Pre-incubation light exposure	-0.02	0.01	-2.23	0.03
Chilling temperature	-0.18	0.05	-3.61	<0.01
Heat treatment	-0.33	0.04	-7.65	<0.01
Incubation light	0.01	0.01	0.64	0.52

The model was performed with a binomial error and logit link function.

**Figure 8:**
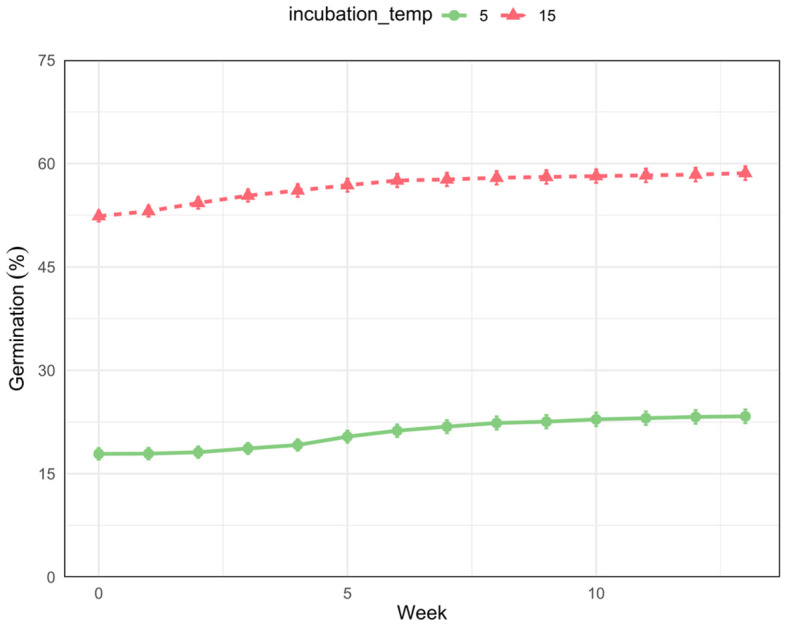
A plot of cumulative percentage seed germination for *Ivesia webberi* seeds incubated under 5/1°C or 15/2°C cold-stratified temperature regimes.

A separate GLMM with incubation light exposure as a random effect (12-hour light vs 24-hour darkness) showed that all pre-incubation treatments and incubation temperatures significantly affected *I. webberi* seed germination (*p* < 0.05; **Table 3**). Seeds have higher germination rates under 15/2°C than 5/1°C incubation temperature (**Figure 8**). Fisher’s Exact test also showed a significant difference in seed germinations between 15/2 °C and 5/1 °C incubation temperatures (χ2 = 39.12, df = 1, *p* < 0.001).

**Table 3:** Results of the generalized linear mixed model for *I. webberi* seed germination subjected to varying pre-incubation light (12-hour light vs 24-hour darkness), either cold moist (1 or2°C), warm dry or warm moist (30/15°C), and either 5/1°C or 15/2°C incubation temperature, while accounting for incubation light exposure (12-hour light exposure or 24-hour darkness) as a random effect.

Factor	Estimates	Standard error	z-value	*P*
Intercept	-1.15	0.07	-17.23	<0.01
Pre-incubation light exposure	-0.02	0.01	-2.19	0.03
Chilling temperature	-0.18	0.05	-3.68	<0.01
Heat treatment	-0.33	0.04	-7.63	<0.01
Incubation temperature	0.04	0.01	7.07	<0.01

The model was performed with a binomial error and logit link function.

A baseline GLMM, accounting for incubation temperature between two experimental phases, showed significant effects of growth hormones and pre-incubation heat treatments on seed germination, while pre-incubation light exposure, pre-incubation chilling temperature, and incubation light exposure have nonsignificant effects on seed germination (**Table 4**). Overall, seeds exposed to growth hormone mixture of higher concentrations of potassium nitrate (5055.5 M; KNO_3_) and gibberellic acid (0.003 M; GA_3_) produced the highest germination rate in the first experiment phase (**Supplementary Table 1**). In the second experiment phase, however, seed germination was greater in higher concentrations of GA_3_ or KNO_3_ exposures, while mixture of both growth hormone mixtures did not increase seed germination (**Supplementary Table 1**). Although seeds treated with growth hormones had the highest percentage of germinations, seeds treated to 4-week pre-incubation chilling and cold-stratified incubation performed equal to or better than many of the hormone-induced germinations in both experimental phases (**Supplementary Table 1**).

**Table 4:** Results of the baseline generalized linear mixed model, with incubation temperature (5/1°C or 15/2°C) as a random effect, for *I. webberi* seed germination subjected to varying pre-incubation light (12-hour light exposure or 24-hour darkness), either cold moist (1 or 2°C), warm dry or warm moist (30/15°C), and varying incubation light exposure (12-hour light exposure or 24-hour darkness).

Factor	Estimates	Standard error	z-value	*P*
Intercept	-1.13	0.14	-7.88	<0.01
Pre-incubation light exposure	-0.02	0.01	-1.90	0.06
Chilling temperature	-0.04	0.05	-0.77	0.44
Heat treatment	-0.12	0.05	-4.07	<0.01
Incubation light exposure	0.01	0.01	0.64	0.52
Gibberellic acid treatment	0.49	0.08	6.00	<0.01
Potassium nitrate treatment	0.37	0.08	4.72	<0.01

The model was performed with a binomial error and logit link function.

### 3.7 Effects of pre-incubation and incubation treatments on mean germination time and synchrony of *I. webberi* seed germination

For the first germination experiment phase (5/1°C), mean germination time was the fastest for pre-incubated heat exposed seeds, followed by hormone-induced germinations and pre-incubated chilled seeds, while the two controls (no pre-incubation treatments) had the slowest germination times (**Supplementary Table 1**). However, germination times were faster for all treatments in the second experiment phase (15/2°C) than in the first phase (**Supplementary Table 1**). We also observed a significant inverse relationship (*r* = -0.41, df = 270, *p* < 0.001) between the number of germinated seeds and mean germination time across all 68 treatments used in this study. Analysis of variance results on both experiment phases showed that all pre-incubation and incubation treatments, except incubation light exposure, significantly influenced germination time (**Table 5**). Similar results were obtained for separate ANOVA tests conducted for the first and second experimental phases (**Supplementary Tables 4**, **5**).

**Table 5:** Effects of pre-incubation (varying light exposure, chilling vs heat treatments), varying incubation light exposure, and incubation temperature, and differing concentrations and mixtures of gibberellic acid and potassium nitrate treatments on time and synchrony of *Ivesia webberi* seed germination under both 5/1°C and 15/2°C incubation temperature, using analysis of variance.

Factor	df	Mean germination time				Synchronization index			
		SS	MSS	F	*P*	SS	MSS	F	*P*
Pre-incubation light	1	4.37	4.37	6.81	<0.01	0.04	0.04	2.05	0.15
Chilling temperature	2	29.44	14.72	22.94	<0.01	1.79	0.90	49.16	<0.01
Heat treatment	2	43.07	21.53	33.56	<0.01	1.58	0.79	43.43	<0.01
Incubation temperature	1	63.41	63.41	98.82	<0.01	1.48	1.48	81.51	<0.01
Incubation light	1	0.02	0.02	0.03	0.87	0.00	0.00	0.00	0.98
Gibberellic acid	2	16.32	8.16	12.71	<0.01	0.52	0.26	14.13	<0.01
Potassium nitrate	2	15.18	7.59	11.83	<0.01	0.70	0.35	19.29	<0.01
Residuals	260	166.85	0.64			4.73	0.02		

Furthermore, we observed synchronized germination only for seeds subjected to pre-incubation heat treatment in the first experimental phase, while greater seed germination synchrony was recorded across all treatments in the second experiment phase (**Supplementary Table 1**). The number of germinated seeds significantly correlated with germination synchrony for both the first and second experiment phases (*r* = 0.47, df = 270, *p* < 0.001), while all treatments, except pre-incubation and incubation light exposures, had significant effects on synchronization index (**Table 5**). Similar results were observed for separate ANOVA tests ran on the first and second experimental phases (**Supplementary Tables 4**, **5**).

## 4 Discussion

### 4.1 Drivers and implications of seed viability in *Ivesia webberi*

Our data showed that *Ivesia webberi* seed viability and potential for germination was the highest within a year of abscission, with reduced viability over longer storage times, suggesting that the seeds have a recalcitrant storage behavior. Recalcitrant seed behavior is common in many perennial plant species (Baldos et al., 2014; Duncan et al., 2019), including Great Basin Desert perennial species (Allen and Nowak, 2008). Seeds that have recalcitrant storage behavior are also likely to form a transient seed bank *in situ* (Guo et al., 1998; Gasparin et al., 2020). Though seed viability loss was rapid within a year, it was not completely lost, suggesting a bet hedging strategy that is also observed in many xeric plant species (Clauss and Venable, 2000). Viability loss in xeric plants is attributed to seed aging due to prolonged light exposure after abscission (Schwember and Bradford, 2011). In addition, this study also showed significant interannual variation in the viability of *I. webberi* seeds. Temporal variability in seed viability may be attributed to various biotic and abiotic factors in the previous or current year that impact flowering, pollination, and seed set (Clauss and Venable, 2000; Yang et al., 2016; Chen J.-Z. et al., 2022). For example, interpopulation variability in seed viability for 2017 collections was significantly associated with heatload and summer AET, indicating the impact of climatic factors on seed viability. This is consistent with *I. webberi* phenology since seed abscission and maturity occur in the summer. In previous studies, climatic stress associated with high ambient temperatures resulted in loss of seed viability, failed seed set, reduced seed quality, and decline in seed vigor and germination (Young et al., 2004; Rang et al., 2011; Rosbakh et al., 2018).

Patch size was not a predictor of seed viability. Although small and isolated populations may produce seeds with relatively low viability due to reduced cross-pollination and higher selfing (Wright et al., 2013; Barrett, 2015; but see Nakayama et al., 2012), we observed that the *I. webberi* population with the lowest estimated density also had relatively high seed viability in the two years of sampling. Contemporary gene flow patterns and time since isolation may play a role in maintaining adaptive genetic variation even under contemporary isolation (Levin, 2012; Borokini et al., 2021a). In a meta-analysis, Baskin and Baskin (2023) showed that seeds from both large and small populations had similar germination rates in more than half of 119 species tested, and they concluded that seed germination was not affected by seed size, population size, genetic diversity or gene flow barriers. Moreover, previous studies showed that small populations of species that exhibit a mixed breeding strategy could still produce a high number of viable seeds (Mayer et al., 1996; Baldwin and Schoen, 2019) by delaying selfing till the end of the flowering season when chances of cross-pollination are reduced (Kalisz and Vogler, 2003; Hildesheim et al., 2019). Interpopulation variability in seed viability will have profound implications on temporal seedling recruitment across sites, which may affect census size and consequently genetic diversity, especially for small and increasingly geographically isolated populations (Hens et al., 2017; Capblancq et al., 2021; Liu et al., 2023).

The tetrazolium test is a standard, but destructive approach that is widely used to screen seeds for viability. Here, we showed that for *Ivesia webberi*, this test can be replaced with equally reliable and non-destructive methods. These results could apply to other achene fruits, although further studies are needed to explore the efficacy of non-destructive methods for other species. The seed x-ray imagery showed that filled, well-developed, and undamaged *I. webberi* seeds could be used as proxy for viability. This finding is supported by previous studies which have also reported the accuracy of seed x-ray images for predicting seed viability (e.g., Costa et al., 2014; Alencar et al., 2016; Gomes et al., 2016; Kim et al., 2017). Though positive seed viability tests do not necessarily result in seed germination, especially for bet hedging species, Riebkes et al. (2015) found significant association among seedling emergence, tetrazolium test, and seed x-ray images for investigating seed viability in three species. Moreover, exposure to radiation from seed x-ray tests was reported to have minimal effect on seed health and germination (Bino et al., 1993; Young et al., 2007). At 690 nm wavelength, non-viable seeds have stronger fluorescent intensity which is associated with higher chlorophyll a content and oxidation, both of which have been linked to reduced tolerance to abiotic stress and reduced germination potential (Cerovic et al., 1999; Dell’Aquila, 2009; Smolikova et al., 2011; Boelt et al., 2018; Li et al., 2019). Viable *I. webberi* seeds had significantly lower spectral values at 690 nm (**Supplementary Table 2**), suggesting the usefulness of multispectral imaging at 690 nm in discriminating between viable and non-viable seeds.

### 4.2 Dormancy release and germination of *Ivesia webberi* seeds

*I. webberi* seed germination was higher and faster in the second experiment phase, characterized by higher incubation and wider cold stratification temperatures, suggesting that warmer winter and spring conditions will both accelerate the seed germination rate and process. This is consistent with field observations that *I. webberi* and other spring emergents regenerate up to two months earlier in milder winters, resulting in dramatic phenological changes. Future climate changes in the Great Basin Desert are predicted to lead to warmer and shorter winters resulting in phenological shifts for winter and spring annuals and perennials (Mondoni et al., 2012, 2015; Tang et al., 2015). This germination result is also congruent with the predictions that increased global temperatures will increase seed germination in higher latitudes and altitudes (De Frenne et al., 2010; Walck et al., 2011; Rosbakh et al., 2018). In addition to phenological shifts, mild winters could result in greater vegetative cover, especially of invasive species (Borokini et al., 2021b). However, if early germination of spring and winter annuals and perennials is followed by winter or spring frost, this may result in seedling death (Walck et al., 2011; Porceddu et al., 2013). It is also important to note that germination in *I. webberi* was associated with myxodiaspory, the release of hydrophilic mucilage from seeds following water imbibition, in hydrated *I. webberi* seeds prior to radicle emergence (Yang et al., 2012; Gorai et al., 2014; Chen Y. et al., 2018). Furthermore, most germination of *I. webberi* seeds occurred within the first two weeks of incubation, which is indicative of relatively “fast” germination syndrome which is associated with survival strategies in highly disturbed habitats such as the Great Basin Desert that are characterized by frequent wildfires and a short growing season (Pierce et al., 2007; Gentili et al., 2013).

We observed *I. webberi* seed germination under varying pre-incubation and incubation treatments, but pre-incubation chilling followed by cold stratification incubation significantly increased *I. webberi* seed germination more than other treatment in both experimental phases. This is consistent with natural conditions under which *I. webberi* seeds germinate – a period of winter cold followed by heat fluxes of late winter and early spring. The effectiveness of pre-incubation chilling and cold stratification incubation on seed germination have been reported for many temperate species (Baskin and Baskin, 2014; Cheng et al., 2022) including achene-producing spring perennials found within the range of *I. webberi* such as *Purshia tridentata* and *Balsamorhiza sagittata* (Young and Evans, 1979; Brown and Allen, 2023). Studies showed that pre-chilling and cold stratification softened seed testa and decreased the concentration of germination inhibitors (Feurtado et al., 2004; Płażek et al., 2018). Light exposure was the only pre-incubation and incubation treatment that had no significant effect on seed germination, indicating that *I. webberi* is a neutral photoblastic species (Baskin and Baskin, 2014). When the seeds abscise, they remain on the soil surface or in surface rock crevices on the soil, therefore, whether the seeds are buried under the snow (total darkness) or chilled on barren cold soil and exposed to periodic winter sunlight, seed germination would occur when cold stratification is initiated. This result is also consistent with studies that show desert plants do not require light for germination (Jurado and Westoby, 1992; Flores et al., 2016) because chilling, water, and cold stratification are more important than light for the germination of spring or early summer annuals and perennials (Rubin and Friedman, 2018; Cheng et al., 2022). Moreover, seeds of desert plants are not likely to be buried under litter or dense canopy, conditions under which light requirements would be adaptive (Fenner and Thompson, 2005).

In this study, all pre-incubation and incubation treatments except light exposures had significant effects on both mean germination time and synchrony. Germination success rate of *I. webberi* seeds is inversely correlated with mean germination time, a proxy for germination speed, but positively associated with synchronization index. For example, in the first experimental phase, pre-incubation chilling treatment produced greater but less synchronized germinations, while faster and synchronized germinations resulted in lower seed germination rates in pre-incubation heat treatments. In the second experiment phase where incubation temperature was higher, seed germination rates were greater, occurred faster and more synchronized in all treatments, indicating the role of incubation temperature on seed germination. Moreover, synchronized germination in higher temperature is a predicted response to more stable environmental conditions (Xu and Du, 2023), while bet hedging strategies are associated with unpredictable environments (Simons, 2011). Thus, a species may exhibit plastic synchronous or asynchronous germination depending on habitat conditions during germination and disturbance frequencies (Xu and Du, 2023).

Seed germination experiments under various dormancy releasing treatments are used to test the regeneration niche hypothesis that plant species occur in habitats where seed germination and seedling establishment are successful (Grubb, 1977; Guerra-Coss et al., 2021; Glison et al., 2023). The ability of *I. webberi* seeds to germinate under various temperature and chemical treatments is indicative of reduced dormancy and a wide regeneration niche, which may be associated with generalist seed germination spectrum where germination occurs rapidly when exposed to conditions that favor dormancy release (Marques et al., 2014; Finch et al., 2019; Fernández-Pascual et al., 2021). Furthermore, successful seed germination under varying conditions may be indicative of asynchronous germination and bet hedging strategies, which have been previously reported for other alpine and subalpine plants as an adaptive response (Liu et al., 2013; Xu and Du, 2023).

### 4.3 *Ivesia webberi* seed embryo morphology and dormancy type

Seed embryo morphology and germination tests can be used to infer the type of dormancy a species exhibits. This knowledge is crucial for successful *ex situ* conservation and optimal seed germination of rare plants. Visual inspection of *I. webberi* seed embryo morphology indicates that the species has a spatulate embryo (Martin, 1946), and can be more specifically classified as “non-endospermic with a spatulate embryo (slightly curved)” (Atwater, 1980). Spatulate seed embryo morphology is common in other rosaceous genera such as *Amelanchier, Coleogyne, Fragaria*, and *Potentilla* (Annette Miller pers. comm.), and lack of endosperm supports field observation that *I. webberi* seeds are not subjected to seed predation or granivory. Species with non-endospermic and spatulate embryos are not mature when they abscise from the plant but require summer heat for maturation, during which period the seed endocarp thickens (Gudin et al., 1990). Increased endocarp thickness in achenes is associated with physiological dormancy as observed in many temperate rosaceous species (Tanowitz et al., 1987; Gudin et al., 1990; Baskin and Baskin, 2014). However, the endocarp in *I. webberi* seeds is permeable to water allowing for dormancy release, as we have shown in the imbibition test.

Spatulate embryo and successful germination of *I. webberi* under variable incubation temperature with or without cold stratification is associated with type-2 nondeep physiological dormancy (Baskin and Baskin, 2004; Shimono and Kudo, 2005; Porceddu et al., 2013). Cold stratification and snowmelt associated with late winter and early spring seasons are required to break physiological dormancy and facilitate seed germination in alpine and sub-alpine plant species (Baskin and Baskin, 2014). The delay of germination until cold stratification and increased warming in late winter or early spring is a reproductive strategy in seeds that exhibit physiological dormancy to prevent autumn germinations thus avoiding the death of seedlings due to freezing winter temperatures (Schwienbacher et al., 2011; Bernareggi et al., 2016; Fernández-Pascual et al., 2021). Significantly reduced seed germination under a warm pre-incubation treatment, which is associated with morphological dormancy, indicates that *I. webberi* seeds do not likely exhibit morphological dormancy.

## 5 Conclusion

We have shown that *Ivesia webberi*, a U.S. federally threatened forb in the Great Basin Desert, exhibits a recalcitrant seed dormancy behavior possibly associated with a transient seed bank, and a mild bet hedging strategy. Seed viability varies temporally, but much less across populations and irrespective of their patch sizes. Viability of *I. webberi* seeds can be reliably monitored using nondestructive testing methods including seed x-ray and multispectral imaging. *I. webberi* seeds exhibit nondeep physiological dormancy; dormancy release is optimal with synchronous germination under warmer cold stratified temperature or growth hormones, while higher, asynchronous germination rate is associated with natural conditions of winter cold period (pre-incubation chilling) followed by spring-like cold stratification incubation. Lack of germination synchrony may indicate bet hedging strategies, which could be a plastic response to the variability of spring conditions in the Great Basin Desert.

The regeneration niche of *I. webberi* is characterized by post-winter temperature increase and water availability from snowmelt or rain, typical of late winter and early spring weather. The timing of seed germination also matches vegetative regeneration of adult *I. webberi* from root caudices, suggesting that the role of cold stratification in the regeneration phenology of *I. webberi* extends also to asexual reproduction. As *I. webberi* has a generalist seed germination behavior, climate change may have profound impacts on the species phenology that could result in earlier germinations, which in turn could increase the vulnerability of seedlings to late-season frost.

## Data Availability

The datasets generated for this study can be found in Knowledge Network for Biocomplexity: doi:10.5063/F1Q52N09.
