# Supplementary material for: Seed biology and regeneration niche of the threatened cold desert perennial Ivesia webberi A. Gray
Source: Front Plant Sci. 2025 May 5;16:1568951. doi: 10.3389/fpls.2025.1568951 (PMC12086156; doi:10.3389/fpls.2025.1568951)
Supplement: Supplementary file 1 [file DataSheet1.docx]

Supplementary Material

**Supplementary Table 1.** Seed germination successes, speed, and synchronization for all 68 pre-incubation and incubation treatments for *Ivesia webberi*

| **#** | **Treatment description** | **Germinated seeds** | **Mean germination time** | **Synchronization index Z** |
| --- | --- | --- | --- | --- |
| 1 | No chill, cold-moist stratified (5/1 ˚C) incubation (12/12 hr.) for 12 weeks | 31 | 5.68 | 2.47 |
| 2 | No chill, cold-moist stratified (5/1 ˚C) incubation (0 hr.) for 12 weeks | 22 | 5.23 | 2.05 |
| 3 | Chill (1 ˚C) for 4 weeks (12/12 hr. photoperiod), cold-moist stratified (5/1 ˚C) incubation (12/12 hr.) for 12 weeks | 36 | 3.34 | 2.25 |
| 4 | Chill (1 ˚C) for 4 weeks (0 hr. photoperiod), cold-moist stratified (5/1 ˚C) incubation (12/12 hr.) for 12 weeks | 14 | 1.96 | 1.40 |
| 5 | Chill (1 ˚C) for 4 weeks (12/12 hr. photoperiod), cold-moist stratified (5/1 ˚C) incubation (0 hr.) for 12 weeks | 14 | 1.88 | 1.51 |
| 6 | Chill (1 ˚C) for 4 weeks (0 hr. photoperiod), cold-moist stratified (5/1 ˚C) incubation (0 hr.) for 12 weeks | 24 | 2.18 | 1.73 |
| 7 | Warm-moist stratification (30/15 ˚C) for 4 weeks (14/10 hr. photoperiod), cold-moist stratified (5/1 ˚C) incubation (0 hr.) for 12 weeks | 14 | 0.30 | 0.89 |
| 8 | Warm-dry stratification (30/15 ˚C) for 4 weeks (14/10 hr. photoperiod), cold-moist stratified (5/1 ˚C) incubation (0 hr.) for 12 weeks | 10 | 1.85 | 1.01 |
| 9 | Warm-moist stratification (30/15 ˚C) for 4 weeks (14/10 hr. photoperiod), chill (1 ˚C) for 4 weeks (12/12 hr. photoperiod), cold-moist stratified (5/1 ˚C) incubation (12/12 hr.) for 12 weeks | 11 | 0.61 | 0.88 |
| 10 | Warm-moist stratification (30/15 ˚C) for 4 weeks (14/10 hr. photoperiod), chill (1 ˚C) for 4 weeks (12/12 hr. photoperiod), cold-moist stratified (5/1 ˚C) incubation (0 hr.) for 12 weeks | 8 | 0.10 | 0.68 |
| 11 | Warm-moist stratification (30/15 ˚C) for 4 weeks (14/10 hr. photoperiod), chill (1 ˚C) for 4 weeks (0 hr. photoperiod), cold-moist stratified (5/1 ˚C) incubation (12/12 hr.) for 12 weeks | 5 | 0.08 | 0.37 |
| 12 | Warm-moist stratification (30/15 ˚C) for 4 weeks (14/10 hr. photoperiod), chill (1 ˚C) for 4 weeks (0 hr. photoperiod), cold-moist stratified (5/1 ˚C) incubation (0 hr.) for 12 weeks | 5 | 0 | 0.44 |
| 13 | Warm-dry stratification (30/15 ˚C) for 4 weeks (14/10 hr. photoperiod), chill (1 ˚C) for 4 weeks (12/12 hr. photoperiod), cold-moist stratified (5/1 ˚C) incubation (12/12 hr.) for 12 weeks | 6 | 0.5 | 0.53 |
| 14 | Warm-dry stratification (30/15 ˚C) for 4 weeks (14/10 hr. photoperiod), chill (1 ˚C) for 4 weeks (12/12 hr. photoperiod), cold-moist stratified (5/1 ˚C) incubation (0 hr.) for 12 weeks | 12 | 0.87 | 0.78 |
| 15 | Warm-dry stratification (30/15 ˚C) for 4 weeks (14/10 hr. photoperiod), chill (1 ˚C) for 4 weeks (0 hr. photoperiod), cold-moist stratified (5/1 ˚C) incubation (12/12 hr.) for 12 weeks | 24 | 1.24 | 1.32 |
| 16 | Warm-dry stratification (30/15 ˚C) for 4 weeks (14/10 hr. photoperiod), chill (1 ˚C) for 4 weeks (0 hr. photoperiod), cold-moist stratified (5/1 ˚C) incubation (0 hr.) for 12 weeks | 24 | 0.73 | 1.21 |
| 17 | Warm-moist stratification (30/15 ˚C) for 4 weeks (14/10 hr. photoperiod), cold-moist stratified (5/1 ˚C) incubation (12/12 hr.) for 12 weeks | 14 | 0 | 0.44 |
| 18 | Warm-dry stratification (30/15 ˚C) for 4 weeks (14/10 hr. photoperiod), cold-moist stratified (5/1 ˚C) incubation (12/12 hr.) for 12 weeks | 13 | 0.70 | 0.51 |
| 19 | Soak seeds in 1000 ppm GA_3_ for 48 hours, cold-moist stratification (5/1 ˚C) for 12 weeks (12/12 hr. photoperiod) | 28 | 1.48 | 1.31 |
| 20 | Soak seeds in 1000 ppm GA_3_ for 48 hours, cold-moist stratification (5/1 ˚C) for 12 weeks (0 hr. photoperiod) | 29 | 1.80 | 1.43 |
| 21 | Soak seeds in 500 ppm GA_3_ for 48 hours, cold-moist stratification (5/1 ˚C) for 12 weeks (12/12 hr. photoperiod) | 29 | 1.84 | 1.50 |
| 22 | Soak seeds in 500 ppm GA_3_ for 48 hours, cold-moist stratification (5/1 ˚C) for 12 weeks (0 hr. photoperiod) | 21 | 1.43 | 1.11 |
| 23 | Soak seeds in 0.1% KNO_3_ for 48 hours, cold-moist stratification (5/1 ˚C) for 12 weeks (12/12 hr. photoperiod) | 25 | 1.64 | 1.19 |
| 24 | Soak seeds in 0.1% KNO_3_ for 48 hours, cold-moist stratification (5/1 ˚C) for 12 weeks (0 hr. photoperiod) | 17 | 1.04 | 0.84 |
| 25 | Soak seeds in 0.5% KNO_3_ for 48 hours, cold-moist stratification (5/1 ˚C) for 12 weeks (12/12 hr. photoperiod) | 27 | 1.77 | 1.23 |
| 26 | Soak seeds in 0.5% KNO_3_ for 48 hours, cold-moist stratification (5/1 ˚C) for 12 weeks (0 hr. photoperiod) | 32 | 1.86 | 1.31 |
| 27 | Soak seeds in 1000 ppm GA_3_ and 0.1% KNO_3_ for 48 hours, cold-moist stratification (5/1 ˚C) for 12 weeks (12/12 hr. photoperiod) | 42 | 1.82 | 1.61 |
| 28 | Soak seeds in 1000 ppm GA_3_ and 0.1% KNO_3_ for 48 hours, cold-moist stratification (5/1 ˚C) for 12 weeks (0 hr. photoperiod) | 35 | 1.56 | 1.28 |
| 29 | Soak seeds in 500 ppm GA_3_ and 0.1% KNO_3_ for 48 hours, cold-moist stratification (5/1 ˚C) for 12 weeks (12/12 hr. photoperiod) | 19 | 0.74 | 0.84 |
| 30 | Soak seeds in 500 ppm GA_3_ and 0.1% KNO_3_ for 48 hours, cold-moist stratification (5/1 ˚C) for 12 weeks (0 hr. photoperiod) | 20 | 1.11 | 0.88 |
| 31 | Soak seeds in 1000 ppm GA_3_ and 0.5% KNO_3_ for 48 hours, cold-moist stratification (5/1 ˚C) for 12 weeks (12/12 hr. photoperiod) | 51 | 1.80 | 1.57 |
| 32 | Soak seeds in 1000 ppm GA_3_ and 0.5% KNO_3_ for 48 hours, cold-moist stratification (5/1 ˚C) for 12 weeks (0 hr. photoperiod) | 44 | 2.02 | 1.52 |
| 33 | Soak seeds in 500 ppm GA_3_ and 0.5% KNO_3_ for 48 hours, cold-moist stratification (5/1 ˚C) for 12 weeks (12/12 hr. photoperiod) | 44 | 1.58 | 1.37 |
| 34 | Soak seeds in 500 ppm GA_3_ and 0.5% KNO_3_ for 48 hours, cold-moist stratification (5/1 ˚C) for 12 weeks (0 hr. photoperiod) | 41 | 1.63 | 1.30 |
| 35 | No chill, cold-moist stratified (15/2 ˚C) incubation (12/12 hr.) for 12 weeks | 27 | 0.61 | 0.92 |
| 36 | No chill, cold-moist stratified (15/2 ˚C) incubation (0 hr.) for 12 weeks | 26 | 0.78 | 0.90 |
| 37 | Chill (2 ˚C) for 4 weeks (12/12 hr. photoperiod), cold-moist stratified (15/2 ˚C) incubation (12/12 hr.) for 12 weeks | 31 | 0.38 | 0.91 |
| 38 | Chill (2 ˚C) for 4 weeks (0 hr. photoperiod), cold-moist stratified (15/2 ˚C) incubation (12/12 hr.) for 12 weeks | 37 | 0.34 | 1.06 |
| 39 | Chill (2 ˚C) for 4 weeks (12/12 hr. photoperiod), cold-moist stratified (15/2 ˚C) incubation (0 hr.) for 12 weeks | 25 | 0.29 | 0.77 |
| 40 | Chill (2 ˚C) for 4 weeks (0 hr. photoperiod), cold-moist stratified (15/2 ˚C) incubation (0 hr.) for 12 weeks | 22 | 0.38 | 0.71 |
| 41 | Warm-moist stratification (30/15 ˚C) for 4 weeks (14/10 hr. photoperiod), cold-moist stratified (15/2 ˚C) incubation (12/12 hr.) for 12 weeks | 19 | 0.06 | 0.59 |
| 42 | Warm-dry stratification (30/15 ˚C) for 4 weeks (14/10 hr. photoperiod), cold-moist stratified (15/2 ˚C) incubation (12/12 hr.) for 12 weeks | 29 | 0.81 | 0.93 |
| 43 | Warm-moist stratification (30/15 ˚C) for 4 weeks (14/10 hr. photoperiod), cold-moist stratified (15/2 ˚C) incubation (0 hr.) for 12 weeks | 30 | 0.19 | 0.68 |
| 44 | Warm-dry stratification (30/15 ˚C) for 4 weeks (14/10 hr. photoperiod), cold-moist stratified (15/2 ˚C) incubation (0 hr.) for 12 weeks | 26 | 0.63 | 0.76 |
| 45 | Warm-moist stratification (30/15 ˚C) for 4 weeks (14/10 hr. photoperiod), chill (2 ˚C) for 4 weeks (12/12 hr. photoperiod), cold-moist stratified (15/2 ˚C) incubation (12/12 hr.) for 12 weeks | 14 | 0.02 | 0.04 |
| 46 | Warm-moist stratification (30/15 ˚C) for 4 weeks (14/10 hr. photoperiod), chill (2 ˚C) for 4 weeks (12/12 hr. photoperiod), cold-moist stratified (15/2 ˚C) incubation (0 hr.) for 12 weeks | 12 | 0.05 | 0.07 |
| 47 | Warm-moist stratification (30/15 ˚C) for 4 weeks (14/10 hr. photoperiod), chill (2 ˚C) for 4 weeks (0 hr. photoperiod), cold-moist stratified (15/2 ˚C) incubation (12/12 hr.) for 12 weeks | 15 | 0.01 | 0.07 |
| 48 | Warm-moist stratification (30/15 ˚C) for 4 weeks (14/10 hr. photoperiod), chill (2 ˚C) for 4 weeks (0 hr. photoperiod), cold-moist stratified (15/2 ˚C) incubation (0 hr.) for 12 weeks | 17 | 0.05 | 0.07 |
| 49 | Warm-dry stratification (30/15 ˚C) for 4 weeks (14/10 hr. photoperiod), chill (2 ˚C) for 4 weeks (12/12 hr. photoperiod), cold-moist stratified (15/2 ˚C) incubation (12/12 hr.) for 12 weeks | 16 | 0.17 | 0.48 |
| 50 | Warm-dry stratification (30/15 ˚C) for 4 weeks (14/10 hr. photoperiod), chill (2 ˚C) for 4 weeks (12/12 hr. photoperiod), cold-moist stratified (15/2 ˚C) incubation (0 hr.) for 12 weeks | 26 | 0.46 | 0.75 |
| 51 | Warm-dry stratification (30/15 ˚C) for 4 weeks (14/10 hr. photoperiod), chill (2 ˚C) for 4 weeks (0 hr. photoperiod), cold-moist stratified (15/2 ˚C) incubation (12/12 hr.) for 12 weeks | 29 | 0.43 | 0.78 |
| 52 | Warm-dry stratification (30/15 ˚C) for 4 weeks (14/10 hr. photoperiod), chill (2 ˚C) for 4 weeks (0 hr. photoperiod), cold-moist stratified (15/2 ˚C) incubation (0 hr.) for 12 weeks | 22 | 0.27 | 0.62 |
| 53 | Soak seeds in 1000 ppm GA_3_ for 48 hours, cold-moist stratification (15/2 ˚C) for 12 weeks (12/12 hr. photoperiod) | 44 | 0.68 | 1.09 |
| 54 | Soak seeds in 1000 ppm GA_3_ for 48 hours, cold-moist stratification (15/2 ˚C) for 12 weeks (0 hr. photoperiod) | 51 | 0.76 | 1.17 |
| 55 | Soak seeds in 500 ppm GA_3_ for 48 hours, cold-moist stratification (15/2 ˚C) for 12 weeks (12/12 hr. photoperiod) | 33 | 0.72 | 0.89 |
| 56 | Soak seeds in 500 ppm GA_3_ for 48 hours, cold-moist stratification (15/2 ˚C) for 12 weeks (0 hr. photoperiod) | 32 | 0.63 | 0.86 |
| 57 | Soak seeds in 0.1% KNO_3_ for 48 hours, cold-moist stratification (15/2 ˚C) for 12 weeks (12/12 hr. photoperiod) | 38 | 0.70 | 0.95 |
| 58 | Soak seeds in 0.1% KNO_3_ for 48 hours, cold-moist stratification (15/2 ˚C) for 12 weeks (0 hr. photoperiod) | 36 | 0.63 | 0.87 |
| 59 | Soak seeds in 0.5% KNO_3_ for 48 hours, cold-moist stratification (15/2 ˚C) for 12 weeks (12/12 hr. photoperiod) | 43 | 0.63 | 1.00 |
| 60 | Soak seeds in 0.5% KNO_3_ for 48 hours, cold-moist stratification (15/2 ˚C) for 12 weeks (0 hr. photoperiod) | 31 | 0.46 | 0.71 |
| 61 | Soak seeds in 1000 ppm GA_3_ and 0.1% KNO_3_ for 48 hours, cold-moist stratification (15/2 ˚C) for 12 weeks (12/12 hr. photoperiod) | 31 | 0.53 | 0.73 |
| 62 | Soak seeds in 1000 ppm GA_3_ and 0.1% KNO_3_ for 48 hours, cold-moist stratification (15/2 ˚C) for 12 weeks (0 hr. photoperiod) | 49 | 0.74 | 1.10 |
| 63 | Soak seeds in 500 ppm GA_3_ and 0.1% KNO_3_ for 48 hours, cold-moist stratification (15/2 ˚C) for 12 weeks (12/12 hr. photoperiod) | 27 | 0.44 | 0.63 |
| 64 | Soak seeds in 500 ppm GA_3_ and 0.1% KNO_3_ for 48 hours, cold-moist stratification (15/2 ˚C) for 12 weeks (0 hr. photoperiod) | 30 | 0.48 | 0.72 |
| 65 | Soak seeds in 1000 ppm GA_3_ and 0.5% KNO_3_ for 48 hours, cold-moist stratification (15/2 ˚C) for 12 weeks (12/12 hr. photoperiod) | 35 | 0.38 | 0.76 |
| 66 | Soak seeds in 1000 ppm GA_3_ and 0.5% KNO_3_ for 48 hours, cold-moist stratification (15/2 ˚C) for 12 weeks (0 hr. photoperiod) | 37 | 0.66 | 0.80 |
| 67 | Soak seeds in 500 ppm GA_3_ and 0.5% KNO_3_ for 48 hours, cold-moist stratification (15/2 ˚C) for 12 weeks (12/12 hr. photoperiod) | 27 | 0.42 | 0.61 |
| 68 | Soak seeds in 500 ppm GA_3_ and 0.5% KNO_3_ for 48 hours, cold-moist stratification (15/2 ˚C) for 12 weeks (0 hr. photoperiod) | 47 | 0.55 | 0.95 |

**Supplementary Table 2.** Results of multivariate multiple logistic regression on the effect of bioclimatic and topographic predictors on the viability of *Ivesia webberi* seeds collected in 2017 and 2018.

| **Factor** | **2017** | | | | **2018** | | | |
| --- | --- | --- | --- | --- | --- | --- | --- | --- |
|  | **Odds ratio^a^** | **Standard error** | **t value** | ***P*** | **Odds ratio^a^** | **Standard error** | **t value** | ***P*** |
| Intercept | 5.69 | 3.58 | 4.35 | 0.01 | 0.14 | 7.90 | -0.25 | 0.81 |
| Summer 2017 AET | 1.05 | 0.01 | 4.49 | 0.01 | - | - | - | - |
| Summer 2018 AET | - | - | - | - | 1.04 | 0.04 | 1.00 | 0.36 |
| Heatload | 0 | 4.12 | -4.17 | 0.01 | 15.57 | 9.10 | 0.30 | 0.78 |
| Cosine aspect | 1.00 | 0.00 | 2.10 | 0.09 | 1.00 | 0.00 | -0.04 | 0.97 |
| Slope | 1.06 | 0.02 | 2.37 | 0.06 | 0.96 | 0.05 | -0.93 | 0.40 |

*^a^Odds ratios were derived as exponent of the raw model estimated coefficients.*

**Supplementary Table 3.** Seed x-ray imagery and multispectral reflectance variables used to construct random forest tree model on *Ivesia webberi* seed viability

| **Predictors** | **Viable seeds** | | **Nonviable seeds** | | **T-test** | **Predictor description** |
| --- | --- | --- | --- | --- | --- | --- |
|  | **Mean±SD** | **Range** | **Mean±SD** | **Range** |  |  |
| Seed area | 3.62±0.65 | 1.05 – 6.25 | 3.79±0.74 | 1.73 – 6.09 | *P* < 0.001 | Computed in mm^2^ from both vertical and horizontal dimensions of seed image |
| Seed x-ray | 0.97±0.18 | 0.00 – 1.00 | 0.53±0.50 | 0.00 – 1.00 | *P* < 0.001 | Binary score of 0 and 1 for unfilled and filled seeds respectively, based on likelihood of presence of seed embryo |
| Multicolor mean 13 | 16.50±4.30 | 8.84 – 29.09 | 18.09±3.95 | 9.65 – 27.75 | *P* < 0.001 | Seed testa spectral reflectance value obtained using 690 nm wavelength |

**Supplementary Table 4.** Effects of pre-incubation (varying light exposure, chilling vs heat treatments), varying incubation light exposure, and differing concentrations and mixtures of gibberellic acid and potassium nitrate treatments on time and synchrony of *Ivesia webberi* seed germination under 5/1 ˚C incubation temperature, using analysis of variance.

| **Factor** | **df** | **Mean germination time** | | | | **Synchronization index** | | | |
| --- | --- | --- | --- | --- | --- | --- | --- | --- | --- |
|  |  | **SS** | **MSS** | **F** | ***P*** | **SS** | **MSS** | **F** | ***P*** |
| Pre-incubation light | 1 | 3.07 | 3.07 | 3.43 | 0.07 | 0.01 | 0.01 | 0.10 | 0.75 |
| Chilling temperature | 1 | 10.21 | 10.21 | 11.38 | <0.01 | 0.01 | 0.01 | 0.11 | 0.75 |
| Heat treatment | 2 | 63.25 | 31.62 | 35.26 | <0.01 | 1.68 | 0.84 | 31.36 | <0.01 |
| Incubation light | 1 | 0.17 | 0.17 | 0.19 | 0.66 | 0.00 | 0.00 | 0.00 | 0.97 |
| Gibberellic acid | 2 | 29.83 | 14.92 | 16.63 | <0.01 | 0.53 | 0.26 | 9.85 | <0.01 |
| Potassium nitrate | 2 | 25.84 | 12.92 | 14.41 | <0.01 | 0.78 | 0.39 | 14.46 | <0.01 |
| Residuals | 266 | 113.02 | 0.90 |  |  | 3.38 | 0.03 |  |  |

**Supplementary Table 5.** Effects of pre-incubation (varying light exposure, chilling vs heat treatments), varying incubation light exposure, and differing concentrations and mixtures of gibberellic acid and potassium nitrate treatments on time and synchrony of *Ivesia webberi* seed germination under 15/2 ˚C incubation temperature, using analysis of variance.

| **Factor** | **df** | **Mean germination time** | | | | **Synchronization index** | | | |
| --- | --- | --- | --- | --- | --- | --- | --- | --- | --- |
|  |  | **SS** | **MSS** | **F** | ***P*** | **SS** | **MSS** | **F** | ***P*** |
| Pre-incubation light | 1 | 1.45 | 1.45 | 30.57 | <0.01 | 0.11 | 0.11 | 20.86 | <0.01 |
| Chilling temperature | 1 | 1.95 | 1.95 | 41.27 | <0.01 | 0.12 | 0.12 | 23.75 | <0.01 |
| Heat treatment | 2 | 2.62 | 1.31 | 27.71 | <0.01 | 0.33 | 0.17 | 32.68 | <0.01 |
| Incubation light | 1 | 0.05 | 0.05 | 1.11 | 0.29 | 0.00 | 0.0 | 0.00 | 0.98 |
| Gibberellic acid | 2 | 0.12 | 0.06 | 1.28 | 0.28 | 0.09 | 0.04 | 8.38 | <0.01 |
| Potassium nitrate | 2 | 0.32 | 0.16 | 3.37 | 0.04 | 0.11 | 0.05 | 10.34 | <0.01 |
| Residuals | 266 | 5.96 | 0.05 |  |  | 0.64 | 0.01 |  |  |


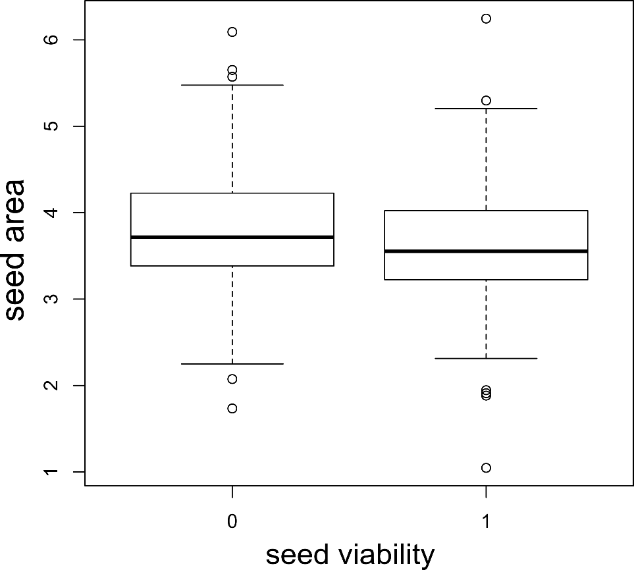


**Supplementary Figure 1.** Box plot showing the relationship between seed area and viability for *Ivesia webberi*. Viability was determined using the tetrazolium test.

**Supplementary Result 1. Is there a relationship between seed area and seed viability?**

We measured the length and width of *I. webberi* seeds (n = 324 seeds for 2017 and n = 441 seeds for 2018) from which seed area was calculated. The measurements were done using VideometerLab 3 instrument (Videometer A/S, Hørsholm, Denmark) at Skyway Analytics LLC as part of the multispectral imaging. Following this, the viability of the seeds was evaluated using the TZ test. To investigate a statistical relationship between seed size and viability, we conducted a logistic regression with 70% of the data and used the remaining 30% for model evaluation. We also investigated interpopulation variability in seed size across *I. webberi* populations using chi-squared test and logistic regression. Logistic regression showed an inverse but significant relationship between seed area and viability for *I. webberi* seeds collected in 2018 (GLM: odds ratio = 0.70, z = -2.49, *P* = 0.01), but not for seeds collected in 2017. Overall, the majority of viable *I. webberi* seeds were relatively small (Figure S1).
